# Case Report: Diabetic ketoacidosis in a patient with Klinefelter syndrome: a rare and complex presentation

**DOI:** 10.3389/fendo.2025.1651488

**Published:** 2026-01-14

**Authors:** Yuwen Wu, Haohua Deng, Jiazhong Sun, Xin Li

**Affiliations:** Department of Endocrinology, Zhongnan Hospital of Wuhan University, Wuhan, Hubei, China

**Keywords:** case report, diabetic ketoacidosis, diagnosis and treatment, hypogonadism, Klinefelter syndrome

## Abstract

**Background:**

Klinefelter syndrome (KS) is a sex chromosome abnormality disease, with a reported incidence of 0.1%–0.2%, while the prevalence of KS in male infertility patients is about 3.1%. Patients with KS are prone to experience metabolic abnormalities over time, such as obesity, diabetes, and metabolic syndrome, along with sexual dysfunction.

**Case presentation:**

This report describes a case in which diabetic ketoacidosis (DKA) developed in an individual with KS. Specifically, the case report concerns a 29-year-old male who presented to our hospital with a 3-day history of headache and poor appetite accompanied by a half-day of nausea and vomiting. After excluding issues with the patient’s digestive system, DKA was suspected, and the patient was admitted to the hospital. Upon abdominal CT, it was noted by chance that the patient had small testicles and a small penis, and an interview with the patient and their family revealed that he had previously been diagnosed with Kallmann syndrome. After extra examination, including chromosome examination, it was revealed that the patient had an extra X chromosome (47, XXY), and Klinefelter syndrome was diagnosed.

**Conclusion:**

This case report provides case experience that can improve physicians’ understanding of Klinefelter syndrome, including the differential diagnosis and treatment of the disease and its further progression, as well as the emergence and treatment of other diseases that may occur in subsequent developments and changes in such patients.

## Introduction

Klinefelter syndrome (KS) is a sex chromosome abnormality disease, with a prevalence of 152 per 100,000 newborn males, with about 25% of all KS males diagnosed postnatally (1). The diagnosis of KS is often delayed due to the wide variability in its clinical manifestations; however, it is usually characterized by small and firm testicles, hypergonadotropic hypogonadism (HH), and azoospermia (2, 3). The early identification of the disorder and the timely initiation of testosterone replacement therapy (TRT) can substantially improve the quality of life of KS patients and prevent additional serious consequences occurring. Low testosterone levels, such as in HH, have been reported to be associated with reduced insulin sensitivity, while promoting the accumulation of visceral fat, and contributing to insulin resistance (4, 5). Individuals with KS have been reported to have a higher incidence of metabolic disorders and autoimmune diseases (6). This case report presents a comprehensive analysis of the disease in a KS patient with a complication of diabetic ketoacidosis (DKA). The paper presents real clinical experience, which the authors hope will promote a greater understanding of KS and support the improved diagnosis and treatment of KS. Note, written informed consent was obtained from the patient for the publication of potentially identifiable images and the clinical details of their case; albeit all the images have been cropped and anonymized to the fullest extent possible to protect the patient’s identity.

## Case presentation

**Figure 1:**
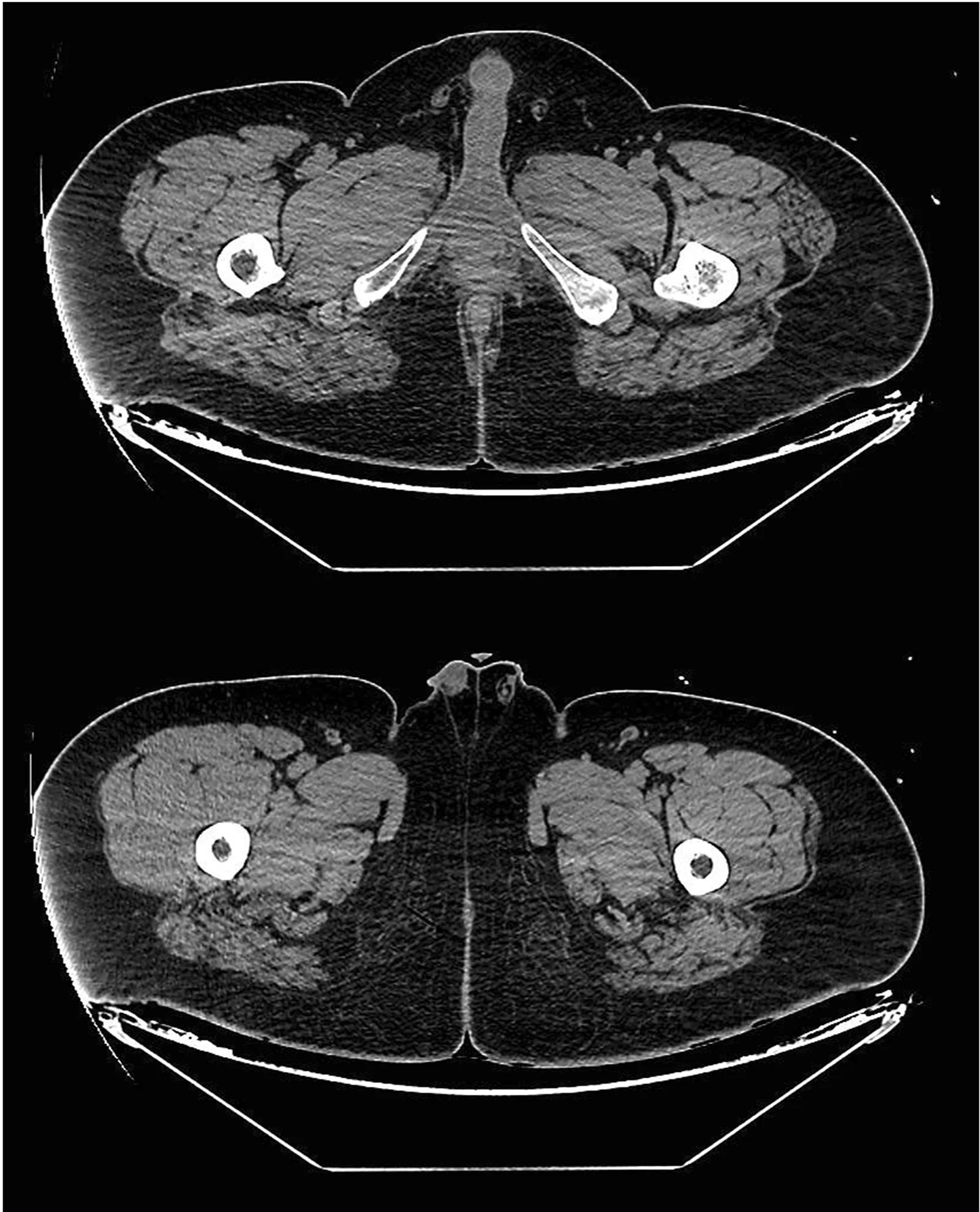
CT scan revealing a small penis and small testicles.

A 29-year-old male presented to Zhongnan Hospital on 26 July, 2023 with a 3-day history of headache and poor appetite, accompanied by a half-day of nausea and vomiting. The patient reported having developed the headache without any obvious cause, and that this was accompanied by fatigue and poor appetite. He initially thought he had a “cold” and rested at home. However, after experiencing nausea and vomiting, including evacuating his stomach contents, accompanied by shortness of breath, he decided to attend the hospital the next day. Routine physical examination was performed at the hospital, and he exhibited the following vital signs: temperature: 37.8 °C; heart rate: 104 BPM; respiration: 18 BPM; blood pressure: 121/65 mmHg; blood oxygen saturation: 98%; height: 177 cm; weight: 98 kg; and BMI: 31.3 kg/m^2^. Extra emergency measurements and tests were then performed as well as arterial blood gas analysis, revealing a pH of 7.1, plasma glucose level of 30.2 mmol/L, and standard base excess (SBE) of -26.9 mmol/L, with normal lipase and amylase. Considering a one-year history of elevated fasting plasma glucose, the patient was admitted to the emergency room for the treatment of suspected DKA. Abdominal CT was performed to detect the source of infection causing their symptoms and assess for pancreatitis, which was considered a possible risk due to the extraordinary infection indicators observed. The CT scan revealed that the patient had a fatty liver, gallstones, small testicles, and a small penis (**Figure 1**).

After admission to the Department of Endocrinology, laboratory blood measurements revealed a ketones level of 3.72 mmol/L, HbA1c of 14.0%, uric acid of 557.8 μmol/L, total cholesterol of 5.53 mmol/L, triglycerides of 12.72 mmol/L, and LDL-c of 4.98 mmol/L. The IAA, GAD, ICA, anti-insulin antibodies, thyroid functions and cortisol (8 AM) levels were all found to be in the normal range. Urine analysis showed strong positive results for glucose and ketones. Following the test results, the patient received continuous electrocardiographic monitoring, oxygen therapy, fluid replacement, intravenous insulin infusion to lower the blood sugar level, maintenance of the water electrolyte balance, acid correction (sodium bicarbonate injection), and lipid-lowering (fenofibrate) and other supportive treatments. After all the aforementioned treatments, the headache, nausea, and vomiting the patient had previously experienced were finally relieved, and the patient’s ketones test was negative. Based on the patient interview, the likely triggers for his condition were identified as a solitary lifestyle and poor dietary control. His work in logistics contributed to irregular eating patterns, daily consumption of sugary drinks, and frequent prolonged outdoor exposure. Additionally, inadequate protection against cold may result in repeated colds and infections.

During hospitalization, the patient exhibited depressive tendencies and was reluctant to engage in conversation. Other family members revealed that the patient had previously been diagnosed with Kallmann syndrome at another hospital due to an abnormal development of the external genitalia. The patient underwent androgen therapy for several months in junior high school, but did not persist with this treatment.

**Figure 2:**
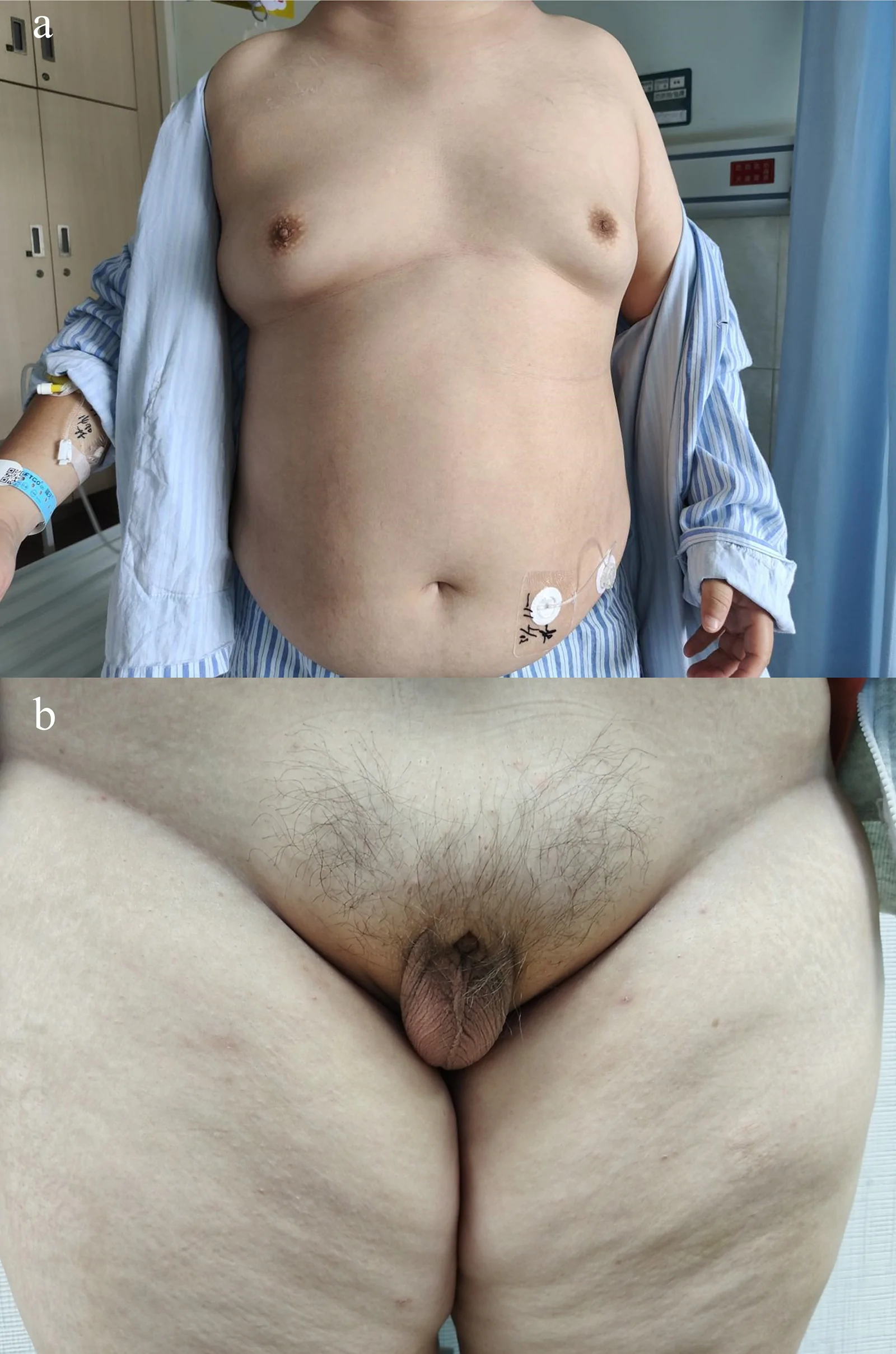
Physical examination revealing breast development **(a)** and a small penis **(b)**.

While the abdominal CT findings (small testicles and penis) and physical examination findings (no underarm hair, gynecomastia, immature development of external genitalia, small scrotum on both sides, see **Figure 2**) gave some indications about the disease, it was decided that more measurements were needed. The patient’s growth hormone, thyroid hormone, and cortisol levels were tested and found to be normal. However, testing of the sex hormones showed some abnormalities. Also, the patient had a luteinizing hormone (LH) level of 21.7 mIU/mL, follicle-stimulating hormone (FSH) level of 20.30 mIU/mL, and total testosterone level of 0.33 ng/mL (**Table 1**), indicating hypergonadotropic hypogonadism. The patient denied having hyposmia or heterosmia. Next, given the prior misdiagnosis of Kallmann syndrome, a brain MRI was performed to rule out pituitary or hypothalamic lesions, and no structural issues were found. Bone density measurements were also performed and the T-scores were within the normal range—1.1 at the lumbar spine, 0.5 at the femur, and 1.2 at the hip. He was subsequently discharged with a plan for regular follow-up to monitor his ongoing fracture risk.

**Table 1:** Lab measurements and test results.

Inspection items	Value	Reference range	Unit
WBC	7.3	4.0–10.0	10^9/L
RBC	5.29	3.5–5.5	10^12/L
Hb	159	120–170	G/L
PLT	173	100–300	10^9/L
glu	30.2	3.9–6.1	mmol/L
FCP	2.682	1.06–4.81	ng/mL
TC	5.53	<5.18	mmol/L
LDL-c	4.98	<3.37	mmol/L
HDL-c	0.55	>1.04	mmol/L
TG	12.72	<1.7	mmol/L
ua	557.8	208–428	μmol/L
HbA1c	14.0	4.0–6.5	%
LH	21.7	1.7–8.6	mIU/mL
FSH	20.3	1.5–12.4	mIU/mL
PRL	2.39	4.04–15.2	ng/mL
TT	0.33	2.49–8.36	ng/mL
E2	33.7	11.4–43.2	pg/mL
cORTISOL (8AM)	14	8.7–22.4	μg/dL
TSH	1.755	0.25–4.3	mIU/L
FT3	2.963	1.92–4.44	pg/mL
FT4	14.32	10.51–22.7	pmol/L

WBC, white blood cells; RBC, red blood cells; Hb, hemoglobin; PLT, platelets; Glu, glucose; FCP, fasting C-peptide; TC, total cholesterol; LDL-c, low-density lipoprotein cholesterol; HDL-c, high-density lipoprotein cholesterol; TG, triglycerides; UA, uric acid; LH, luteinizing hormone; FSH, follicle-stimulating hormone; PRL, prolactin; TT, total testosterone; E2, estradiol; TSH, thyroid stimulation hormone.

Finally, cytogenetic analysis of 20 G-banded metaphases was performed, and confirmed a 47, XXY karyotype in all the cells examined, and Klinefelter syndrome (KS) was diagnosed and formally confirmed. As treatment, supplementary androgen therapy was recommended.

The diagnoses at discharge included Klinefelter syndrome, diabetic ketoacidosis, hyperlipidemia, hyperuricemia, fatty liver, and gallstones.

At 3 month follow-up, the patient weighed 99 kg with a self-monitoring blood glucose (SMBG) level of 8–11 mmol/L and lipid parameters in the normal range. Again the patient reiterated that he did not wish to undergo androgen therapy despite receiving counseling regarding the potential metabolic and systemic benefits of testosterone replacement therapy (TRT), including an explanation of the distinction between TRT, which can offer various benefits but does not reliably restore fertility in 47,XXY individuals, such as him, and fertility options, such as sperm retrieval and assisted reproduction.

## Discussion

Klinefelter syndrome is caused by a failure of the sex chromosomes to separate during meiosis of the sperm or testicles (a few cases also occur during the mitosis of fertilized testicles) (7). It is characterized by hyalinization and fibrosis of the seminiferous tubules (the parts responsible for sperm production) and hypergonadotropic hypogonadism and may include various clinical manifestations, such as increased testicular consistency, azoospermia, gynecomastia, sexual dysfunction, and infertility, as well as glucose and lipid metabolism disorders, obesity, osteoporosis, decreased muscle strength, cognitive impairment, psychological problems (8) and an increased prevalence of pancreatic and thyroid autoimmunity issues (9, 10).

The presented case underscores the critical importance of an early and accurate diagnosis of KS. In this case, the patient had been misdiagnosed and inadequately treated for years, which likely contributed to the development of severe metabolic complications, including DKA. The delayed diagnosis of KS prevents the early initiation of TRT, which is known can improve body composition, insulin sensitivity, and overall metabolic health and could have potentially mitigated the complications that the present patient experienced (3, 4, 11).

This patient’s case was exacerbated as he had initially been misdiagnosed with Kallmann syndrome. Although Klinefelter syndrome and Kallmann syndrome both show symptoms of hypogonadism, the etiology of the diseases and the other symptoms are dramatically different (12). To further aid clinicians in distinguishing between these two disorders, we herein summarize their key differential features as follows: Kallmann syndrome is characterized by hypogonadotropic hypogonadism and frequent olfactory deficits, whereas Klinefelter syndrome typically exhibits hypergonadotropic hypogonadism with preserved olfactory function. Additionally, Kallmann syndrome is a genetic disorder that affects the hypothalamus, and is characterized by a delay or absence of puberty and an impaired sense of smell. The hypothalamus is responsible for releasing the gonadotropin-releasing hormone (GnRH), which stimulates the pituitary gland to release FSH and LH. Without GnRH, the testes or ovaries do not receive the signal to mature, leading to hypogonadism. Anosmia occurs due to the underdevelopment of the olfactory bulbs. The levels of FSH and LH (hypogonadotropic or hypergonadotropic), combined with the lack of a sense of smell, are the key points for a differential diagnosis between Kallmann syndrome and Klinefelter syndrome (13).

In this case, the patient was admitted to the hospital with a suspicion of diabetic ketoacidosis (DKA). The development of DKA in this patient appears to be a consequence of prolonged, undiagnosed diabetes mellitus, itself likely exacerbated by the insulin resistance associated with chronic hypogonadism in KS. The delayed diagnosis of KS in this patient and the consequent absence of TRT likely contributed to poor long-term glycemic control, ultimately culminating in acute metabolic decompensation. The pathogenesis of the metabolic changes associated with KS remains incompletely understood. Furthermore, the type and severity of diabetes in KS are not identical, suggesting that multiple factors may be involved in the development of diabetes in such patients. Insulin resistance may be an important factor in diabetes development and a characteristic of KS. Breyer et al. reported that insulin binding to erythrocytes was reduced in KS patients without diabetes (14). Additionally, testosterone deficiency has been recognized to have direct and indirect impacts on the metabolism. Insulin resistance has been consistently detected in patients with KS, with a negative correlation reported between the plasma testosterone concentration and insulin resistance (15). The pathophysiological link between hypogonadism and diabetic ketoacidosis in KS may be explained by several mechanisms, including testosterone deficiency, which is known to promote visceral adiposity and insulin resistance, which can exacerbate glycemic dyscontrol (3, 4). Furthermore, a low testosterone level may impair pancreatic β-cell function and reduce insulin secretion (16). These metabolic disturbances, combined with potential delays in KS diagnosis and treatment, likely contribute to an increased risk of acute complications, such as DKA. It is important to distinguish classic 47,XXY Klinefelter syndrome from high-grade sex chromosome aneuploidies (e.g., 48,XXYY; 48,XXXY; 49,XXXXY), which are now recognized as distinct clinical entities with more complex phenotypes and higher risks of neurodevelopmental and systemic involvement (9).

DKA is associated with a significant increase in HbA1c levels in diabetes patients. In the present case, the patient’s HbA1c level at admission was 14.0%, which is considered relatively high in clinical practice. Studies have suggested that a 10.4%–16.9% HbA1c range for individuals with DKA is an initial manifestation of diabetes. Furthermore, testosterone deficiency in KS patients may contribute to poor glycemic control and insulin resistance, potentially increasing the risk of DKA. However, due to the frequent underdiagnosis of KS, the true prevalence of DKA in these patients remains unclear.

In the present case, the patient had previously received short-term androgen treatment at another hospital during their junior high school, but the specific formulation used was not known. Following that treatment, no significant improvement was noted. The patient also stated that he had never experienced ejaculation, including nocturnal emissions. Following an information review and communication with relevant personnel, he believed that he had lost fertility and rejected the option of undergoing TRT, despite it being recommended to him many times due to various reasons. The patient was reluctant to initiate TRT due to his belief that his secondary sexual characteristics would not further develop and that he would be unable to father healthy children, while his unstable employment and income also caused him to have concerns about the affordability of long-term treatment. We recommended he undergo semen analysis and counseling to investigate his fertility options; however, despite multiple attempts at counseling about the benefits of TRT, he continued to decline such treatment.

We recommend semen analysis and counseling regarding assisted reproductive options for such patients. Additionally, patients should be encouraged to participate in counseling to facilitate psychological adjustment, with the long-term aim of improving their confidence in TRT to encourage their acceptance of such therapy. Follow-up with a urology specialist should also be arranged to evaluate the potential for partial fertility assessment.

This case suggests the need for a reframing of the association between KS and DKA; whereby, rather than viewing KS as a potential risk factor of DKA, it would be more accurate to consider the syndrome as a significant predisposing context that creates a state of heightened metabolic vulnerability. The chronic testosterone deficiency inherent in KS promotes visceral adiposity, contributes to insulin resistance, and may impair pancreatic β-cell function. This underlying metabolic dysregulation, especially when compounded by a delay in diagnosis, which frequently occurs in such patients and which prevents the initiation of protective testosterone therapy, sets the scene for the development of poorly controlled type 2 diabetes. The eventual DKA episode that occurs then represents an acute compensation based on the long-standing, unaddressed risk factors.

The primary clinical lessons of the present case study are threefold. First, in males presenting with DKA, especially those with an atypical phenotype, clinicians should actively consider and screen for underlying hypogonadism, including KS. Second, once KS is diagnosed, it must be recognized as a condition warranting proactive and lifelong metabolic surveillance, including regular glycemic monitoring. Finally, and most importantly, the early introduction of TRT should be advocated, not just for its androgen-specific effects, but as a fundamental strategy to improve metabolic health and reduce the risk of severe diabetic complications.

## Conclusion

Klinefelter syndrome should be considered in males presenting with hypogonadism and metabolic complications such as DKA. The differentiation between Klinefelter syndrome and Kallmann syndrome is essential due to differences in their etiology and management. The present case highlights that patients with Klinefelter syndrome are at risk of developing severe metabolic complications, including diabetic ketoacidosis, likely exacerbated by testosterone deficiency and insulin resistance. A timely diagnosis and consideration of TRT may not only address the risk of hypogonadism but also improve the patients’ metabolic health and reduce the risk of acute diabetic complications. This case underscores the need for increased clinical vigilance, comprehensive hormonal evaluation, and multidisciplinary management in patients with Klinefelter syndrome to prevent serious metabolic decompensation. Furthermore, this case underscores that effective management must address the psychosocial barriers that may be evident in such patients, including stigma and unrealistic expectations, and that can prevent such patients from accepting lifelong therapy.

## Data Availability

The raw data supporting the conclusions of this article will be made available by the authors, without undue reservation.

## References

[1] Gravholt CH Chang S Wallentin M Fedder J Moore P Skakkebæk A . Klinefelter syndrome: integrating genetics, neuropsychology, and endocrinology. Endocr Rev. (2018) 39:389–423. doi: 10.1210/er.2017-00212 29438472

[2] Lanfranco F Kamischke A Zitzmann M Nieschlag E . Klinefelter’s syndrome. Lancet. (2004) 364:273–83. doi: 10.1016/s0140-6736(04)16678-6 15262106

[3] Hovnik T Zitnik E Avbelj Stefanija M Bertok S Sedej K Bancic Silva V . An adolescent boy with klinefelter syndrome and 47,Xxy/46,Xx mosaicism: case report and review of literature. Genes. (2022) 13:744. doi: 10.3390/genes13050744 35627128 PMC9141365

[4] Lucas-Herald AK Aksglaede L Caspersen ID Ahmed SF Carlomagno F Isidori AM . New horizons in klinefelter syndrome: current evidence, gaps, and research priorities. Endocr Rev. (2025) 46:447–78. doi: 10.1210/endrev/bnaf005 39932051

[5] Zitzmann M Sansone A Jannini EA Jones H Ferlin A Lunenfeld B . Recommendations for diagnosis and treatment of the aging male with klinefelter syndrome. Aging Male. (2025) 28:2519035. doi: 10.1080/13685538.2025.2519035 40536882

[6] Bennani G Zahri S Khaldi M Benouna G Drighil A Habbal R . Unusual coexistence of restrictive heart disease and kallmann syndrome: A case report. Egypt Heart J. (2024) 76:50. doi: 10.1186/s43044-024-00479-1 38635120 PMC11026310

[7] Bonomi M Rochira V Pasquali D Balercia G Jannini EA Ferlin A . Klinefelter syndrome (Ks): genetics, clinical phenotype and hypogonadism. J Endocrinol Invest. (2017) 40:123–34. doi: 10.1007/s40618-016-0541-6 27644703 PMC5269463

[8] Blackburn J Ramakrishnan A Graham C Bambang K Sriranglingam U Senniappan S . Klinefelter syndrome: A review. Clin Endocrinol (Oxf). (2025) 102:565–73. doi: 10.1111/cen.15200 39806878

[9] Spaziani M Carlomagno F Tarantino C Angelini F Paparella R Tarani L . From klinefelter syndrome to high grade aneuploidies: expanding the gene-dosage effect of supernumerary X chromosomes. J Clin Endocrinol Metab. (2024) 109:e1564–e73. doi: 10.1210/clinem/dgad730 38193351 PMC11244175

[10] Carlomagno F Minnetti M Angelini F Pofi R Sbardella E Spaziani M . Altered thyroid feedback loop in klinefelter syndrome: from infancy through the transition to adulthood. J Clin Endocrinol Metab. (2023) 108:e1329–e40. doi: 10.1210/clinem/dgad281 37216911 PMC10584011

[11] Butler G Srirangalingam U Faithfull J Sangster P Senniappan S Mitchell R . Klinefelter syndrome: going beyond the diagnosis. Arch Dis Child. (2023) 108:166–71. doi: 10.1136/archdischild-2020-320831 35948402 PMC7614197

[12] Stamou MI Georgopoulos NA . Kallmann syndrome: phenotype and genotype of hypogonadotropic hypogonadism. Metabolism. (2018) 86:124–34. doi: 10.1016/j.metabol.2017.10.012 29108899 PMC5934335

[13] Richard-Eaglin A . Male and female hypogonadism. Nurs Clin North Am. (2018) 53:395–405. doi: 10.1016/j.cnur.2018.04.006 30100005

[14] Breyer D Cvitković P Skrabalo Z Pedersen O Rocić B . Decreased insulin binding to erythrocytes in subjects with klinefelter’s syndrome. J Clin Endocrinol Metab. (1981) 53:654–5. doi: 10.1210/jcem-53-3-654 6790562

[15] Pei D Sheu WH Jeng CY Liao WK Fuh MM . Insulin resistance in patients with Klinefelter’s syndrome and idiopathic gonadotropin deficiency. J Formos Med Assoc. (1998) 97(8):534–40. 9747063

[16] Navarro G Xu W Jacobson DA Wicksteed B Allard C Zhang G . Extranuclear actions of the androgen receptor enhance glucosestimulated insulin secretion in the male. Cell Metab. (2016) 23(5):837–51. doi: 10.1016/j.cmet.2016.03.015 27133133 PMC4864089

