# Correction: Case Report: Diabetic ketoacidosis in a patient with Klinefelter syndrome: a rare and complex presentation

**DOI:** 10.3389/fendo.2026.1986450

**Published:** 2026-10-08

**Authors:** Yuwen Wu, Haohua Deng, Jiazhong Sun, Xin Li

**Affiliations:** Department of Endocrinology, Zhongnan Hospital of Wuhan University, Wuhan, Hubei, China

**Keywords:** case report, diabetic ketoacidosis, diagnosis and treatment, hypogonadism, Klinefelter syndrome

Reference 15 was erroneously given as “Clark D, Kago T, Sahota K, Rashid T, Yap T. Gender Identity in Klinefelter Syndrome: A Patient-Centered Approach to Treatment. *Ann Med* (2024) 56(1):2406447. Epub 20241009. doi: 10.1080/07853890.2024.2406447”. It should be “Pei D, Sheu WH, Jeng CY, Liao WK, Fuh MM. Insulin resistance in patients with Klinefelter’s syndrome and idiopathic gonadotropin deficiency. J Formos Med Assoc. (1998) 97(8):534–540. PMID: 9747063.”

The second citation for Reference for 15 should be reference 16 and these are the corresponding details of the reference: “Navarro G, Xu W, Jacobson DA, Wicksteed B, Allard C, Zhang G, et al. Extranuclear actions of the androgen receptor enhance glucose-stimulated insulin secretion in the male. Cell Metab. (2016) 23(5):837–851. doi: 10.1016/j.cmet.2016.03.015”.

The original version of this article has been updated.

